# Grape pomace as a natural source of antimicrobial agents for food preservation

**DOI:** 10.3389/fnut.2025.1650450

**Published:** 2025-08-01

**Authors:** Micaela Galante, María Emilia Brassesco, Carollyne Maragoni Santos, Carolina Beres, Ana Elizabeth Cavalcante Fai, Ignacio Cabezudo

**Affiliations:** ^1^Laboratorio de Investigación, Desarrollo y Evaluación de Alimentos (LIDEA), Facultad de Ciencias Bioquímicas y Farmacéuticas (FBioyF), Universidad Nacional de Rosario (UNR) y Consejo Nacional de Investigaciones Científicas y Técnicas (CONICET), Rosario, Argentina; ^2^Universidade Católica Portuguesa, CBQF - Centro de Biotecnologia e Química Fina – Laboratório Associado, Escola Superior de Biotecnologia, Rua Diogo Botelho, Porto, Portugal; ^3^Food and Nutrition Graduate Program, Federal University of Rio de Janeiro State (UNIRIO), Rio de Janeiro, Brazil; ^4^Department of Basic and Experimental Nutrition, Institute of Nutrition, Rio de Janeiro State University (UERJ), Rio de Janeiro, Brazil; ^5^Farmacognosia, FBioyF, UNR y CONICET, Rosario, Argentina

**Keywords:** grape by-products, antimicrobial activity, clean label, phenolic compounds, foodborne pathogens

## Abstract

Grape pomace, a by-product of winemaking, has emerged as a promising source of natural antimicrobial compounds for food applications. In response to increasing concerns regarding foodborne illnesses and consumer demand for clean-label products, its valorization represents a sustainable approach to enhance food safety and shelf life. This review combines a bibliometric analysis with a critical examination of the scientific literature. The bibliometric analysis identifies leading authors, institutions, countries, and research trends related to the use of grape pomace in food preservation. The literature review summarizes extraction techniques and antimicrobial evaluations. Grape pomace is rich in phenolic compounds with demonstrated antibacterial and antifungal activity. The antimicrobial effectiveness depends on factors such as grape variety, extraction method, polyphenol profile, and target microorganisms. Its incorporation into food productsincluding meat, dairy, and beverageshas shown promising results. Additionally, bioactive extracts have been applied in edible films, coatings, and active packaging to inhibit microbial growth and prolong shelf life. The evidence supports the potential of grape pomace as an effective antimicrobial food additive. However, challenges remain, including the need for standardized extraction protocols, deeper understanding of antimicrobial mechanisms, and comprehensive safety and efficacy evaluations in real food systems. Addressing these gaps is essential to facilitate the development of innovative food preservation strategies based on grape pomace bioactives.

## 1 Introduction

In the face of growing environmental concerns, the utilization of by-products from the agricultural industry is an important way to reduce waste, conserve resources and drive the transition to a circular and sustainable food system (1, 2). Within this context, the wine industry, despite its significant economic importance, is a major contributor to agro-industrial residues. The main by-product of the winemaking process is grape pomace (GP), a lignocellulosic matrix composed of grape pulp, seeds, peel, and stalks. According to the International Organisation of Vine and Wine (3), global grape production reached approximately 80.1 million metric tons in 2022, with nearly half of this volume—around 37.3 million tons—allocated to winemaking, including must and juice production. Based on the estimated pomace yield, global GP generation ranges from 7.5 to 11.2 million metric tons annually. On average, for every ton of grapes processed, about 20% becomes waste, primarily as GP (4). The inadequate management of these residues can lead to various negative environmental impacts (5). These include water pollution due to the washing of residues and the leaching of organic compounds, soil degradation from the accumulation of large volumes of organic material (6).

Recognizing these negative environmental impacts and the need for more sustainable production, the valorization of GP emerges as a crucial strategy that also presents a significant opportunity to obtain high-value-added compounds (2). As a rich source of bioactive compounds, many applications of GP in the food, cosmetic and pharmaceutical industry have been demonstrated (7). Its complex matrix is particularly abundant in polyphenols, tocopherols, and various macro- and micronutrients, which have been associated with multiple health-promoting properties, including antimicrobial, antiviral, antioxidant, anti-inflammatory, and anti-aging effects (2, 7). Particularly, phenolic compounds exhibit significant antimicrobial activity by disrupting microbial cell membranes and interfering with vital cellular processes. GP is particularly rich in these bioactive molecules, including phenolic acids, flavonols, flavanols, anthocyanins, tannins and stilbenes. The composition and concentration of the phenolic fraction in GP are influenced by several factors, including grape variety, agronomic practices, pre- and post-harvest conditions, and vinification techniques (7, 8).

In light of the growing challenges faced by the food industry in ensuring microbial food safety, GP is a promising natural alternative to synthetic preservatives (9). Foodborne pathogens pose a significant threat to public health, generating substantial economic costs and eroding trust in the food supply (10) traditional preservation methods, involving synthetic preservatives, are facing growing scrutiny from consumers seeking more natural and healthier alternatives (7). This increasing preference for naturally derived ingredients stems from the potential adverse health effects associated with synthetic preservatives. Moreover, there is a general trend toward more sustainable and “clean label” lifestyles. Consequently, the research and development of effective preservation strategies based on natural sources have become a crucial priority for the food industry and public health (11). This review aims to explore the potential of grape pomace as a natural antimicrobial agent for food preservation by integrating a bibliometric approach and providing an overview of its bioactive compounds, primarily phenolic compounds. It also discusses traditional and advanced extraction and encapsulation techniques, antimicrobial activity, applications in food and packaging, and selected patents to highlight technological advances and innovation opportunities for GP valorization, in alignment with circular economy principles.

## 2 Five-year bibliometric analysis of grape pomace as a natural antimicrobial for food preservation

### 2.1 Research strategy and selection criteria for scientific articles

The analysis of reports on GP and its components (peel, seeds, stalks) as a natural source of antimicrobial bioactive compounds was conducted using the bibliometric review method as a search strategy and data selection criteria (12). The review was conducted using the Scopus and Web of Science (WoS) databases. These databases were queried on January 27, 2025, and results published between 2021 and 2025 were considered. To ensure a thorough assessment of the literature, not only GP, but also its individual components were included in this bibliometric analysis to identify specific research trends and application patterns.

The data used for this study were extracted with the following descriptors: (i) Scopus: TITLE-ABS-KEY and subject area (Agricultural and Biological Sciences OR Immunology and Microbiology OR Materials Science): (grape AND (peel OR seed OR resid* OR waste OR agroindustrial OR by-product OR pomace)) AND (antimicrobial OR fungici* OR antibacteri* OR antifungi*) AND (polyphenol* OR phenol* OR flavon* OR anthocyanin* OR phenolic*); (ii) WoS: TOPIC and web of science categories (Food Science technology OR Microbiology OR Polymer Science OR Materials Science OR Biothecnology applied microbiology OR Nutrition Dietetics): (grape AND (peel OR seed OR resid* OR waste OR agroindustrial OR by-product OR pomace)) AND (antimicrobial OR fungici* OR antibacteri* OR antifungi*) AND (polyphenol* OR phenol* OR flavon* OR anthocyanin* OR phenolic*).

**Figure 1:**
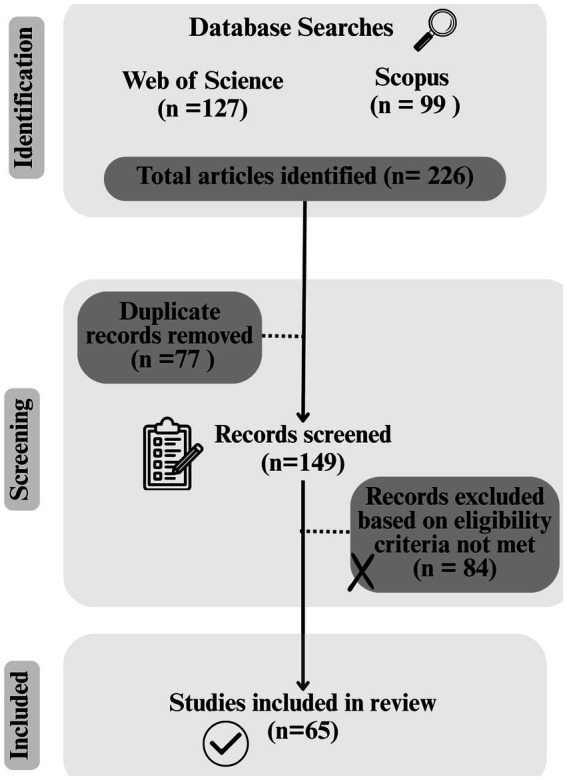
PRISMA flowchart of the bibliometric review on grape pomace as a natural antimicrobial for food preservation.

Then, a review of the title, abstract and keywords was conducted to determine the relevance of the articles to be selected for further analysis. The inclusion criteria were as follows: (i) research articles; (ii) full text in English, either subscription or open access; (iii) scientific articles targeting grape pomace or other grape residues as a natural source of antimicrobials for food preservation; and (iv) studies with data on antimicrobial activity. The exclusion criteria were: (i) review articles, book chapters, theses, dissertations, abstracts, and papers presented in seminars or symposia; (ii) studies on grape pomace or its components that did not include antimicrobial activity data; and (iii) articles on grapes and not on grape pomace or its components. The selected articles that met the inclusion criteria were organized in a spreadsheet program for better data management. A literature review was created considering the previously selected articles (Figure 1).

### 2.2 Bibliometric analysis and scientific performance

**Figure 2:**
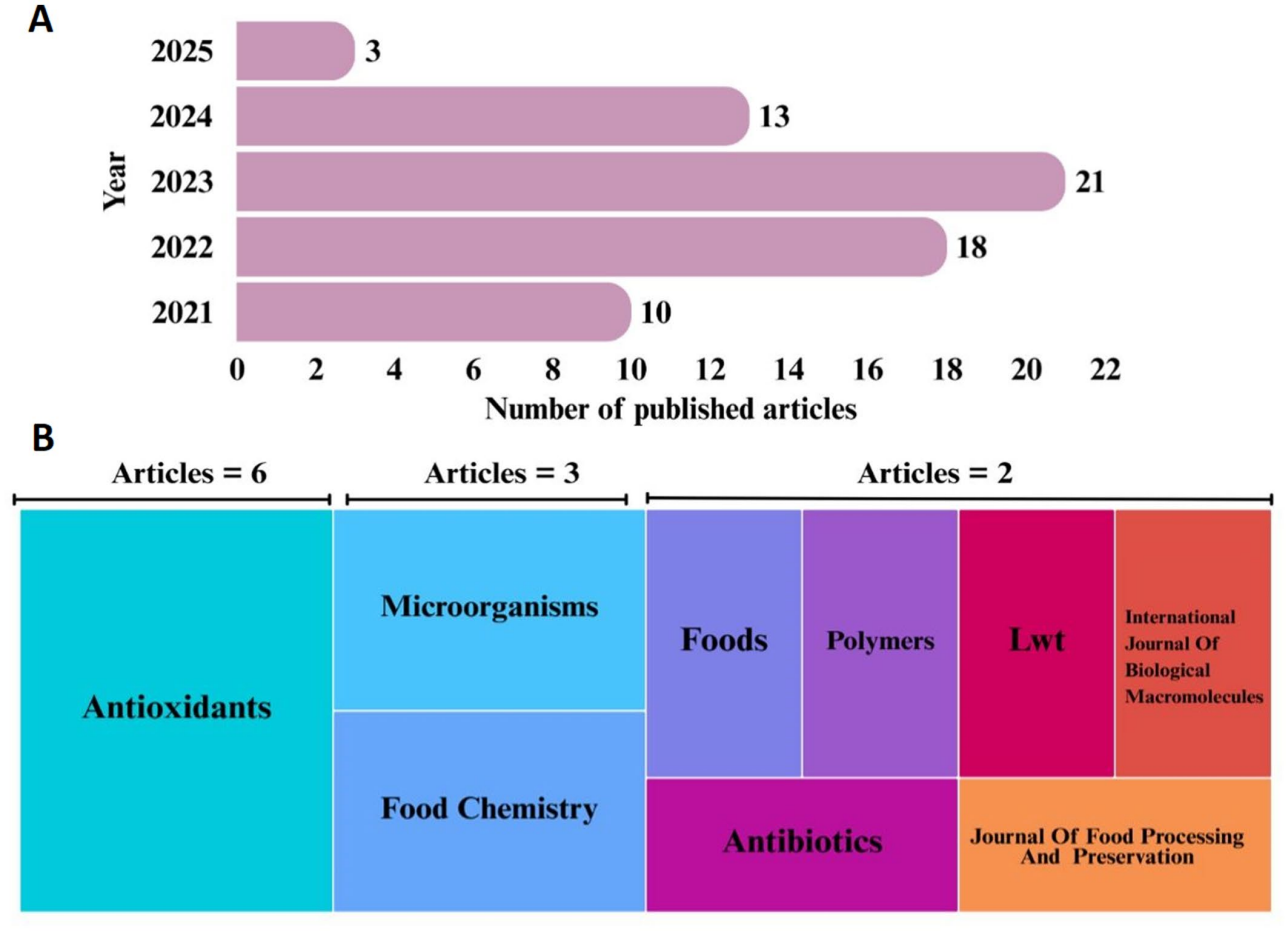
**(A)** Number of documents published between 2021 and 2025 and **(B)** Journals with publications on grape pomace and its components (peel, seeds, stalks) as a natural source of antimicrobial bioactive compounds. Analysis was carried out in the period from 2021 to 2025 only considering research articles.

The keyword search yielded 127 and 99 documents in WoS and Scopus, respectively. After the exclusion of duplicate citations and the outcome of the screening phase, 65 studies were selected to be included in the final analysis and discussion of the results. From 2021 to 2023, the number of articles published on GP and its components as a natural source of antimicrobial bioactive compounds increased (Figure 2A). Most of the selected articles were published in 2023 (*n* = 21), with a decrease in publications observed in 2024 (*n* = 13). The years selected for this search resulted in the following number of published articles: 10 articles in 2021, 18 articles in 2022 and 3 articles in 2025.

Several scientific journals have published research articles on GP and its components as a natural source of antimicrobial bioactive compounds (Figure 2B). The scientific journals in which articles were published as part of this review were identified. Antioxidants (*n* = 6), Microorganisms (*n* = 3), Food Chemistry (*n* = 3), Polymers (*n* = 2), LWT-Food Science and Technology (*n* = 2), International Journal of Biological Macromolecules (*n* = 2), Antibiotics (*n* = 2), Journal of Food Processing and Preservation (*n* = 2) and Foods (*n* = 2) are the main journals with more than one publication on this topic in the above mentioned period.

**Figure 3:**
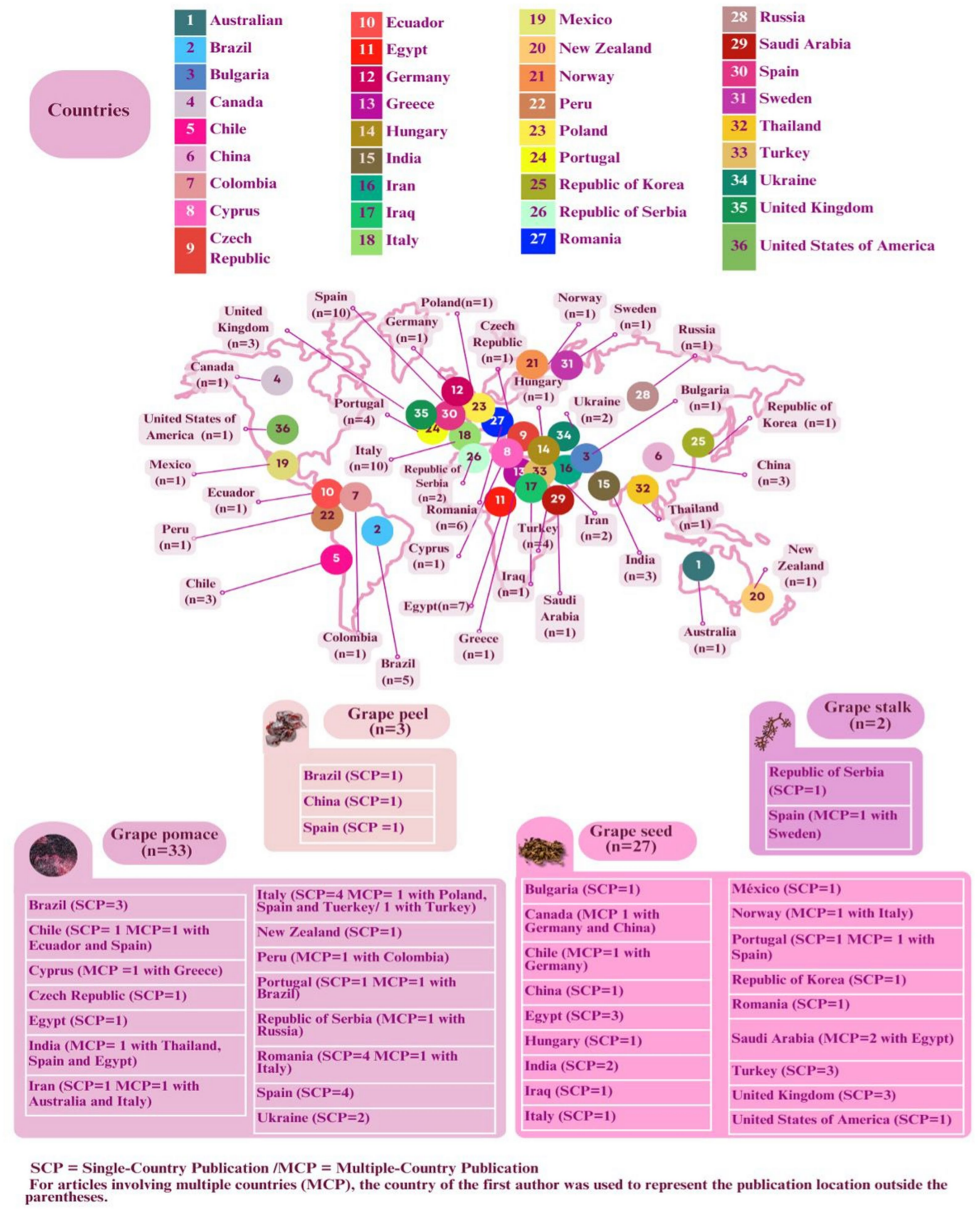
Grape pomace (GP) and its components used as antimicrobial agents and the respective countries in which the studies were conducted. SCP (single-country publications): publications authored by researchers from a single country; MCP (multi-country publications): publications resulting from collaboration among authors affiliated with institutions in different countries.

Figure 3 shows the GP and its components used as well as the respective countries in which the studies were carried out. Thirty seven countries have published on them as a natural source of antimicrobial compounds. The concept of single-country publications (SCP) refers to articles authored by researchers from the same country, while multi-country publications (MCP) indicate international collaboration between authors from different countries. This study identified 48 SCP and 17 MCP and showed that Italy (5 SCP and 5 MCP) and Spain (5 SCPs and 5 MCP) have the most publications on this topic. Egypt (4 SCP and 3 MCP), Romania (5 SCP and 1 MCP), Brazil (4 SCP and 1 MCP) and Portugal (2 SCP and 2 MCP) are the other countries that published the most articles on this topic between 2021 and 2025.

The most important grape by-products used in the selected articles are GP (*n* = 33) and grape seeds (*n* = 27). The use of GP as a potential antimicrobial agent is expected to improve the utilization of this waste at the local level. This is particularly important considering that the major wine-producing countries such as Italy (5.4 million tons in 2022), Spain (3.6 million tons in 2022) and Portugal (666 thousand tons in 2022) (3) account for 53% (*n* = 16) of the selected articles with GP between 2021 and 2025. In addition, it is important to utilize the waste in countries such as Brazil, the 14th largest grape producer, with an estimated loss rate of 8.3% and Egypt, the 13th largest producer, with a loss rate of approximately 9% (3).

To accurately evaluate the antimicrobial potential highlighted by this research, standardized methodologies are essential. In this context, the most frequently used methods for determining microbial inhibitory effects in the selected articles are the agar/disk diffusion test (*n* = 29) and the activity minimum inhibitory concentration (MIC) determination by microdilution technique (*n* = 23). These tests are useful to confirm microbial inhibitory activity over a short period of time. However, they have the limitation that they cannot capture the exact quantitative inactivation dynamics of the microorganism under investigation. Therefore, it is not possible to use only such results for the robust design of an industrial or clinical application (13). The analysis of the selected articles revealed a preponderance of studies on antibacterial activity (*n* = 44), followed by the evaluation of antimicrobial activity against both fungi and bacteria (*n* = 18), while only a few studies exclusively investigated antifungal activity (*n* = 3). Most studies analyzed a combination of Gram-positive and Gram-negative bacteria (*n* = 53). The most important Gram-positive bacteria analyzed were *Staphylococcus aureus* (*n* = 42), while among the Gram-negative bacteria *Escherichia coli* was analyzed most frequently (*n* = 52). Among the fungi, *Candida albicans* was the predominant microorganism (*n* = 11). These cases are developed in Section 4.

This bibliometric overview not only highlights the increasing scientific interest in GP but also underscores the crucial role of its bioactive compounds in driving this research focus. The following section will delve into the specific antimicrobial compounds found in GP.

## 3 Antimicrobial compounds from grape pomace

**Figure 4:**
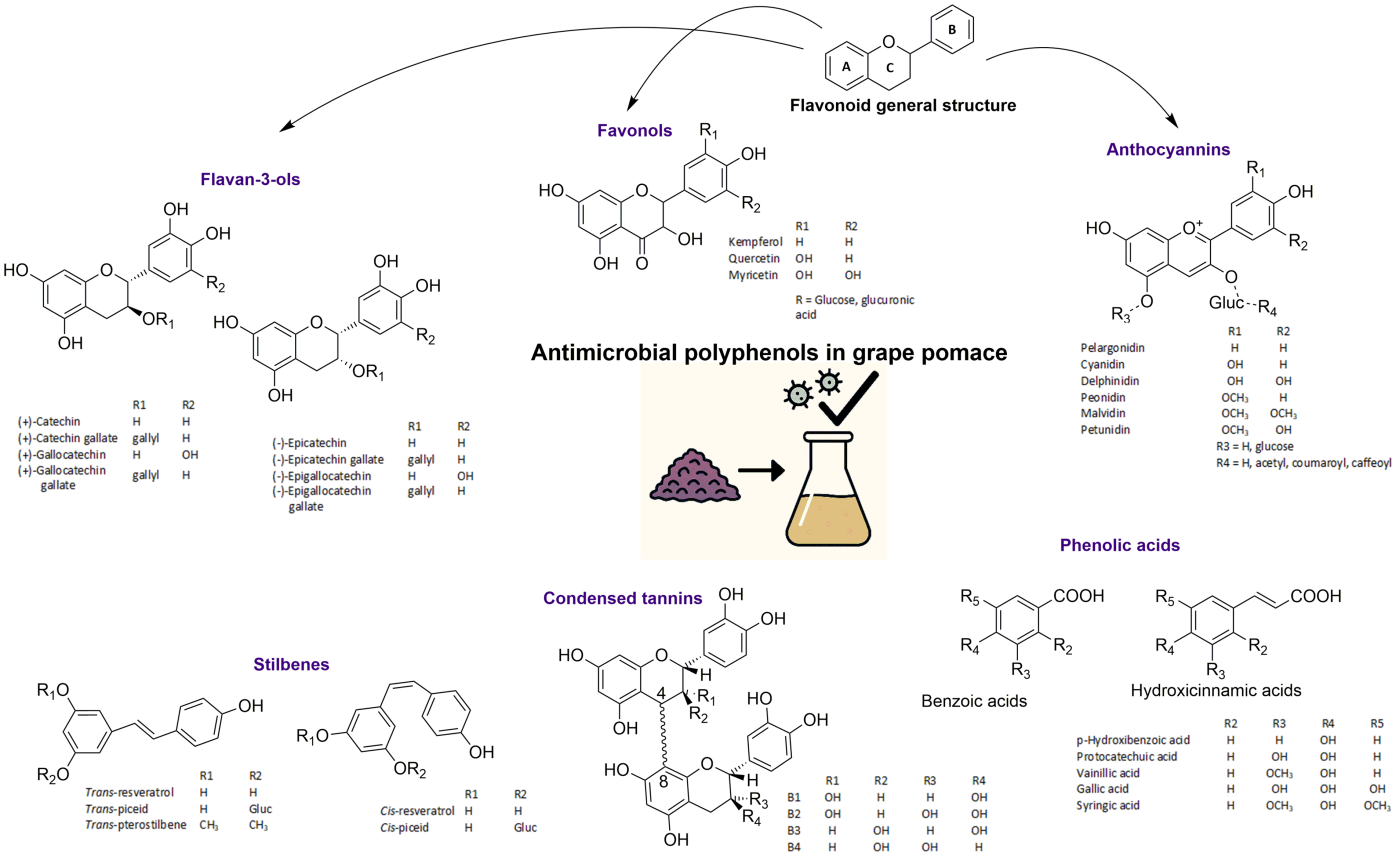
Main phenolic compounds found in grape pomece (GP).

Recent studies have shifted from merely characterizing the composition of GP to elucidating its specific modes of antimicrobial action and identifying the compounds most responsible for this activity. A growing body of evidence highlights that polyphenolic fractions—especially flavonoids, flavanols, and phenolic acids—exert selective inhibitory effects on foodborne pathogens and spoilage microorganisms (5, 14). Beyond polyphenols, GP also contains organic acids, dietary fibers, and minor bioactives such as tocopherols, which may act synergistically to modulate microbial growth (15). Notably, GP is a rich source of these various phenolic compounds (2), Figure 4. Their efficacy depends not only on concentration but also on the extraction methods used and their physicochemical interaction with microbial structures and pathways.

### 3.1 Polyphenols

#### 3.1.1 Flavonoids

Flavonoids, the most abundant polyphenols in human diets and widely distributed throughout the plant kingdom, are characterized by a fundamental C6-C3-C6 structural framework (16). This structure consists of two phenyl rings (A and B) linked by a three-carbon bridge that typically forms a central pyran ring (C) (8), Figure 4. In GP, the primary flavonoid subclasses present in significant amounts are flavan-3-ols (often referred to as flavanols), anthocyanins, and flavonols (16). Other flavonoid groups like flavanones and flavones are generally present in limited concentrations (16, 17).

##### 3.1.1.1 Flavan-3-ols

Flavan-3-ols are flavonoid compounds that significantly influence the nutritional and sensory attributes of grapes and their derivatives, characterized chemically by a hydroxyl group (–OH) attached to the third carbon atom of the C ring, as shown in Figure 4. Flavan-3-ols, including monomers like catechin and epicatechin, and their polymers (condensed tannins or proanthocyanidins), are prevalent in grape seeds and skins, with seeds typically showing higher concentrations (18, 19). Numerous studies highlight their antimicrobial efficacy. Flavan-3-ol-rich extracts from grape seeds and pomace exhibit significant antibacterial activity against *Staphylococcus aureus* (2, 20, 21). Oligomeric proanthocyanidins from grape seeds protect against *Escherichia coli* and *Salmonella enterica* ser. Typhimurium (22), while other extracts inhibit *Bacillus cereus* and *Pseudomonas syringae pv. actinidiae* (23). Flavan-3-ols have also demonstrated broad-spectrum inhibition against spoilage and pathogenic bacteria in food applications, such as ovine stretched cheese (19), and are effective against *Listeria monocytogenes* (13). Beyond antibacterial effects, hydroalcoholic extracts have shown antimycotic activity against *Trichophyton rubrum* and *Arthroderma crocatum* (8). Furthermore, extracts with epicatechin and catechin can inhibit quorum sensing in bacteria (24), and flavan-3-ols contribute to the antibacterial potential of silver nanoparticles (25) and impart antimicrobial properties to active packaging films (26).

##### 3.1.1.2 Flavonols

The presence of flavonols in grape pomace contributes to its overall bioactivity. Quercetin, a prominent flavonol found in grape marc extracts, has demonstrated activity against *Staphylococcus aureus*, indicating the potential of specific phenolic compounds from grape marc for antimicrobial applications (27). Extracts from grape marc, seeds, and stalks, rich in polyphenols including quercetin 3-O-glucuronide and quercetin 3-*O*-glucopyranoside, effectively inhibited the development of mycotoxigenic fungi such as *Aspergillus flavus*, *A. carbonarius*, and *Fusarium graminearum*, and reduced mycotoxin production, suggesting their potential as biofungicides (28). Red GP extracts from the Cabernet variety, containing kaempferol and quercetin alongside other polyphenols, demonstrated antibacterial activity against *Staphylococcus aureus*, *Bacillus subtilis*, and *Pseudomonas fluorescens* (29). Furthermore, the presence of flavonols in red GP powder of the Nero d’Avola cultivar demonstrated a broad inhibition spectrum against spoilage and pathogenic bacteria (19).

##### 3.1.1.3 Anthocyanins

Anthocyanins, the flavonoid pigments responsible for red, blue, and purple hues in grapes and red wines, are primarily glycosylated forms of anthocyanidins (30), shown in Figure 4. Their types and concentrations depend on grape variety and environmental factors (11). Predominantly located in grape skins as 3-*O*-*β*-glucosides (5), these pigments possess significant biological functions, including antimicrobial activities (23). GP retains considerable amounts of these valuable anthocyanins, with delphinidin, cyanidin, petunidin, pelargonidin, peonidin, and malvidin 3-*O*-glucosides and their derivatives being most prevalent (7). Notably, GP extracts rich in anthocyanins demonstrate inhibition against foodborne pathogens like *Bacillus cereus* and *Pseudomonas syringae* pv. actinidiae (23), and various other pathogenic microorganisms (17). Anthocyanins also contribute to the antibacterial potential of silver nanoparticles (25), preserve goat meat by inhibiting spoilage bacteria through binding to bacterial DNA gyrase (31), and exhibit strong antibacterial activity against drug-resistant *Klebsiella* spp. and *Acinetobacter baumannii* complex (32). Extracts with diverse anthocyanins have shown antibacterial activity against susceptible and multidrug-resistant *Escherichia coli* strains (33).

#### 3.1.2 Non-flavonoids

##### 3.1.2.1 Stilbenes

Stilbenes, with resveratrol and its derivatives being prominent examples, are a class of phenolic compounds (Figure 4), well-recognized for their significant antioxidant and anti-inflammatory properties. Stilbenes have demonstrated notable antimicrobial activity, as suggested by studies on aqueous extracts of grape pomace which inhibited the growth of pathogenic microorganisms (17). This same study also indicated potential synergistic effects when these grape pomace extracts were combined with a probiotic, further highlighting the complex bioactivity of the extract (17). Furthermore, specific oligomeric stilbenes present in grapevine byproduct extracts, particularly those derived from stems and roots, such as ampelopsin A and cyphostemmin B, have exhibited substantial antifungal properties against *Fusarium graminearum* (34). This antifungal activity is significant as these compounds were shown to reduce fungal growth and consequently the contamination of cereal crops by mycotoxins (34).

##### 3.1.2.2 Phenolic acids

Beyond flavonoids, GP is a significant source of phenolic acids, which are categorized into hydroxybenzoic and hydroxycinnamic acids (Figure 4). Acid hydrolysis of wine grape marcs (red, pink, and white) yielded hydrolysates exhibiting marked antifungal activity against *Fusarium oxysporum*, an effect attributed to the presence of specific phenolic acids such as gallic acid, hydroxybenzoic acid, vanillic acid, and p-coumaric acid (35). Methanol-ethanol extracts of *Vitis vinifera* seeds, containing epicatechin, catechin, and p-hydroxybenzoic acid identified as major constituents, exhibited antibacterial activity against both Gram-positive and Gram-negative strains and also inhibited quorum sensing-dependent virulence factors in *Pseudomonas aeruginosa* and *Chromobacterium violaceum*, suggesting a synergistic action (24). Grape seed extracts of *Vitis vinifera*, characterized by the presence of 21 phenolic acids and 16 flavonoids identified through HPLC/DAD analysis, demonstrated significant bactericidal and fungicidal effects (36). Red grape pomace extracts from the Cabernet variety, obtained via supercritical carbon dioxide extraction and containing polyphenols such as gallic acid, chlorogenic acid, and ferulic acid, displayed antibacterial activity against *Staphylococcus aureus*, *Bacillus subtilis*, and *Pseudomonas fluorescens* (29). The incorporation of red grape pomace powder (GPP) of the Nero d’Avola cultivar., containing phenolic acids, into ovine stretched cheese enhanced the protein content and potentially provided a broad inhibition spectrum against spoilage and pathogenic bacteria (19). Furthermore, hydroxycinnamic acids, including caffeic acid and gallic acid, contribute to the overall bioactive profile of GP (25).

##### 3.1.2.3 Tannins

GP also contains significant amounts of tannins, both condensed and hydrolyzable, known for their astringency and antimicrobial effects. Tannin extracts from grape peels exhibit bacteriostatic activity against *E. coli* and *L. innocua* (37). The diverse bioactivities of GP extracts, including those from Syrah grape skin (5) and Montepulciano d’Abruzzo (11), are partly attributed to their tannin content. Comparative analyses also highlight the antimicrobial potential of other phenolic compounds like catechin oligomers found in grape seeds (2).

### 3.2 Non-polyphenol antimicrobial bioactive compounds

Beyond polyphenols, grape pomace contains a range of other antimicrobial constituents that act through complementary mechanisms, broadening its antimicrobial spectrum.

Organic acids, such as tartaric and malic acids, are abundant in grape skins and seeds and contribute significantly to microbial inhibition by lowering environmental pH. This acidification denatures bacterial enzymes, impairs nutrient uptake, and interferes with membrane potential. De Andrade et al. (5) demonstrated that Syrah grape skin residues, rich in these acids, effectively suppressed spoilage bacteria in food matrices.

Vitamin C (ascorbic acid), while present in smaller quantities, acts as a pro-oxidant under stress conditions, enhancing polyphenol-mediated antimicrobial activity. Gómez-Mejía et al. (2) showed that vitamin C combined with GP polyphenols potentiated DNA fragmentation and protein oxidation, indicating synergistic bacterial inactivation.

Dietary fibers, including cellulose, hemicellulose, and lignin, also contribute indirectly to antimicrobial defense. These fibers serve as physical barriers that limit microbial adhesion and biofilm development on surfaces. Additionally, during fermentation in the gut, fiber fractions selectively modulate microbiota, suppressing pathogens and supporting beneficial taxa (15, 35). Lignin-derived phenolics released during microbial degradation may also exert bacteriostatic effects.

### 3.3 Mechanisms of antimicrobial action

GP-derived compounds exert antimicrobial effects through multiple, interrelated mechanisms. The antimicrobial potential of these polyphenols is strongly influenced by their structural features, such as the number and position of hydroxyl and methoxy groups, the presence of prenyl substituents and their ability to delocalize *π*-electrons. These features determine their amphipathic nature and their ability to interact with microbial membranes and intracellular targets (38, 39). A primary mechanism of action is the disruption of microbial membranes. Polyphenols interact with phospholipid bilayers and membrane-associated proteins, impairing membrane integrity, increasing permeability and leading to leakage of intracellular contents. An example of this are anthocyanin-rich extracts that cause membrane damage in *Escherichia coli* and *Staphylococcus aureus* (7) and catechins that alter membrane fluidity (40, 41).

In parallel, these bioactive compounds also disrupt enzymes and metabolic processes. Polyphenols can inhibit key enzymes such as ATP synthase and thus disrupt energy metabolism and stop ATP production (11). In addition, studies have shown that they can block the replication and transcription of nucleic acids by inhibiting bacterial DNA gyrase and topoisomerase (31, 40).

Another important antimicrobial mechanism is the inhibition of quorum sensing (QS) and biofilm formation. Polyphenols act as QS inhibitors by interfering with autoinducer signaling pathways and preventing the expression of virulence factors and biofilm-related genes. For example, GP extracts have been shown to inhibit biofilm formation by *Listeria monocytogenes* and *Salmonella* spp. (35).

In addition, specific compounds such as stilbenes and ellagic acid can interfere with bacterial cell division. These polyphenolic components can target FtsZ proteins or DNA polymerases and thus stop proliferation (14, 42). Beyond direct cellular interference, polyphenols also exert chelation of metal ions, especially essential metals such as iron and zinc, depriving microbes of important cofactors for metabolic enzymes (2, 43).

A final important mechanism is the generation of oxidative stress. Certain polyphenols, including resveratrol and ellagic acid, can promote the intracellular accumulation of reactive oxygen species (ROS), leading to oxidative damage to lipids, proteins and DNA. This pro-oxidant activity has been observed in several microbial systems and contributes to cell death pathways (11, 14, 42).

Taken together, these multifaceted effects, membrane disruption, enzyme inhibition, suppression of quorum sensing, impairment of cell division, metal chelation and ROS generation explain the strong and broad antimicrobial efficacy of GP bioactives. These multifunctional mechanisms are closely related to the structural diversity of polyphenolic compounds, which enables them to act simultaneously on multiple microbial targets. Their multifunctionality and synergy with other components make them promising tools for natural food preservation strategies (38, 39, 44).

Comprehensive reviews by Rossi (38), Lobiuc (39), Ecevit (44), and Takó (45) provide detailed discussions on the mechanisms of action and microbial responses related to the antimicrobial potential of polyphenols, further supporting the importance of these compounds for food safety and preservation.

## 4 Valorization of grape pomace: extraction, antimicrobial properties, and encapsulation of bioactive compounds

In the scientific literature, several approaches can be identified for its valorization. Some authors use the whole residue without further processing (46, 47), while others extract bioactive compounds from the bulk material (8, 17, 25, 48–53) or fractionate it into skins, lees, seeds, stems and stalks for targeted extraction (1, 36). In addition, several studies employ commercial grape seed extracts (13, 18, 54–62) or commercial grape skin extract (63), and in some cases, the focus is on oil extraction from the seeds (64). This section discusses the main strategies reported for the valorization of grape pomace, with a particular focus on extraction techniques used to recover bioactive compounds and the analytical methods employed to quantify their antimicrobial properties.

### 4.1 Extraction strategies

Numerous methods have been developed for the extraction of bioactive compounds from complex plant matrices. These range from conventional techniques such as solvent maceration (65, 66) or acid hydrolysis to more advanced methodologies that require specialized equipment, including ultrasound-assisted extraction (UAE) (53, 67), microwave-assisted extraction (MAE) (11), and supercritical fluid extraction (SFE) (1, 29). The quantification of extracted compounds typically involves the Folin–Ciocalteu assay for total phenolic content (TPC) (4, 5, 48) and the colorimetric determination of flavonoids via aluminum chloride complexation (4, 65). Results are commonly expressed in milligrams of gallic acid equivalents (GAE) or rutin/quercetin equivalents per milliliter of extract or gram of dry plant material. In some studies, total anthocyanin content was also evaluated by the pH differential method (4, 65). High-performance liquid chromatography (HPLC) is widely used (33, 68, 69) as well as ultra-high-performance liquid chromatography (UPLC) (4, 17, 70). Some studies further include mass spectrometry for the identification of individual polyphenols (1, 23, 33, 71).

Several authors have employed experimental design approaches to optimize extraction conditions (7, 33). Table 1 provides an overview of the extraction methods reported in the literature for isolating bioactive compounds from GP or its derived fractions. It also details the specific bioactive compounds identified in the resulting extracts, where such identification was conducted by the authors.

**Table 1:** Summarizes the main studies evaluating the antimicrobial potential of bioactive compounds obtained from grape pomace by products.

Vegetal source	Extraction method	Identified bioactive compounds	Antimicrobial determination method	Microorganisms used in the antimicrobial assays	Reference
Winemaking grape by products	Acid hydrolysis treatment	n.d	Agar dilution method	*Fusarium oxysporum* and *Alternaria* spp.	(35)
Grape pomace	Maceration Solvent: absolute ethanol	Punicalagin and Ellagic Acid	Well diffusion assay	*Listeria monocytogenes, Staphylococcus aureus, Enterococcus faecalis,* and *Escherichia coli*	(48)
Grape seeds	Maceration Solvent: water and 95% ethanol solution	n.d	Microdilution method and Gene Expression Test	*Staphylococcus aureus*	(81)
Grape pomace	Maceration Solvent: water and water- ethanol solution	n.d	Microbiological tests	*Escherichia coli, Bacillus megaterium, Listeria monocytogenes, Lactiplantibacillus plantarum*	(17)
Grape seeds	Maceration Solvent: ethanol 90%	n.d	Disk Diffusion Assays	*Escherichia coli, Salmonella typhium, Staphylococcus aureus, Bacillus subtilis,* and *Pseudomonas fluroscens*	(82)
		Gallic acid, Coffeic acid, Procyanidin, Procyanidin; Catechin, Procyanidin -, Procyanidin, Epicatechin, Procyanidin, Procyanidin, Procyanidin, Procyanidin, Procyanidin, Epicatechin	disk diffusion method	*Bacillus subtilis, Staphylococcus aureus, Escherichia coli, Pseudomonas aeruginosa* *Mucor, Candida albicans*	(69)
Grape pomace	Maceration Solvent: ethanol 85.5%	n.d	Disk diffusion method	*S. aureus, E. coli,* and *C. albicans*	(66)
Grape Pomace: stems, seeds, and peels	Maceration Solvent: ethanol 80%	Pyrogallol; Gallic acid; 4-Amino-benzoic acid; Protocatechuic acid; Catechin; Catechol; Caffeine; p -Hydroxy benzoic acid; Chlorogenic acid; Vanillic acid; Caffeic acid; p -Coumaric acid; Ferulic acid; Iso-ferulic acid; *α*-Coumaric acid; Benzoic acid; Ellagic acid; Coumarin; 3,4,5-Methoxy-cinnamic acid; Cinnamic acid; Salicylic acid; Apigenin-6-arabinose-8-galactose; Apigenin-6-rhamnose-8-glucose; Naringin; Hesperidin; Rutin; Apigenin-7-O- neohesperidoside; Kaempferol-3,7-dirhamnoside; Quercitrin; Apigenin-7-O-glucoside; Acacetin-7- neohesperidoside; Quercetin; Naringenin; Hesperetin; Kaempferol; Rhamnetin; Apigenin	Agar well diffusion assay and microdilution method	*Staphylococcus aureus, Bacillus subtilis (B. subtilis, Bacillus megaterium, Escherichia coli, Pseudomonas aeruginosa, Saccharomyces cerevisiae, Candida Albicans*	(36)
Grape pomace: skin and seeds		gallic acid, caftaric acid, hydroxytyrosol and catechin	Dilution method	*Arthroderma crocatum, Arthroderma gypseum, Arthroderma quadrifidum, Trichophyton menta grophytes, Trichophyton mentagrophytes, Tricho phyton rubrum, Trichophyton rubrum, Trichophyton tonsurans*	(8)
Grape seed		Oxalic acid, Quinic acid, Malic acid, Fumaric acid, *α*-tocopherol, *β*-tocopherol, γ-tocopherol, δ-tocopherol	Microdilution method and the rapid p-iodonitrotetrazolium chloride colorimetric assay	*Escherichia coli, Klebsiella pneumoniae,* *Morganella morganii, Pseudomonas aeruginosa, Neisseria gonorrhoeae, Proteus mirabilis*	(2)
Grape seedless pomace and seeds		Gallic acid, Pyragallol, 4-aminobenzoic, Catchine Chlorogenic, Catechol, Epicatechol, Caffeine, p-hydroxybenzoic acid, Caffic, Vanillic, Coumaric, Ferulic, Iso ferulic, Ellagic, Vanilla α-coumaric, Benzoic, 2,4-Dimethoxycinnamic, Coumarine, Salycillic Cinnamic	Agar well diffusion assay/Microdilution Technique	*Staphylococcus Aureus, Bacillus cereus, Bacillus subtilis, Pseudomonas aeruginosa, Klebsiella pneumonia, Escherichia coli, Salmonella typhimurium, Aspergillus niger, Candida albicans*	(80)
Grape Pomace seed	Maceration Solvent: ethanol 70%	Gallic acid: Gallic acid glucoside; Procyanidin B1; Procyanidin B3; (+)-Catechin; Procyanidin B2; (−)-Epicatechin; Procyanidin C1	Agar disk diffusion assay and microdilution method	*S. aureus, E. coli*, and *B. cereus*	(21)
Red grape seeds		Gallic acid; Chlorogenic acid, Catechin; Methyl gallate; Syringic acid, Ellagic acid, Naringenin	n.d	n.d	(94)
Grape pomace flour		n.d	Agar Well Diffusion Assays	*Aeromonas hydrophila, A. salmonicida, Edwardsiella ictaluri, E. tarda, Flavobacterium columnaris, F. psychrophilum, Vibrio anguillarum, V. harveyi, and V. vulnificus.*	(14)
Grape pomace	Maceration solvent: ethanol 60%	Anthocyanin: malvidin derivates (malvidin-3-*O*-(6-acetyl)-glucoside, malvidin-3-*O*-glucoside, malvidin-3-*O*-(6-caffeoyl)-glucoside, and malvidin-3-*O*-(6-coumaroyl)-glucoside); Peonidin 3-*O*-glucoside; Delphinidin-3-*O*-(6-caffeoyl)-glucoside Flavonols: myricetin-3-*O*-galactoside, quercetin-3-*O*-glucoside, Myricetin-3-galactoside; Myricetin-3-*O*-glucuronide, quercetin, catechin and epicatechin	Disk Diffusion Assays	*Bacillus subtilis, Bacillus cereus, Escherichia coli, Staphylococcus aureus, Salmonella enteritidis, Pseudomonas syringae* pv. *actinidiae* and *Listeria monocytogenes*	(23)
	Maceration Solvent: ethanol 50%	Caffeic acid; Chlorogenic; p-Coumaric aci; rutin; Hyperoside; Isoquercetin; Cyanidin-glucoside; Petunidin-glucoside; Malvidin-glucoside;	Microdilution method	*Listeria monocytogenes, Bacillus subtilis, Pseudomonas aeruginosa, Proteus mirabilis, Salmonella enteritidis, Enterococcus faecalis, Staphylococcus aureus,* and *Escherichia coli*	(32)
		n.d	Direct surface inoculation assay	*Escherichia coli, Salmonella Typhymurium,* and *Listeria monocytogenes*	(90)
Grape pomace: skins and seeds	Maceration Solvent: acetone 70%	n.d	Bacterial growth inhibition assay	*B. subtilis, Escherichia coli*	(50)
Grape seed	Maceration (solvent: 75% acetone solution)	n.d	Macro and Micro-Dilution Method	*F. necrophorum sub*sp. *Necrophorum, F. necrophorum sub*sp. *Funduliforme, Salmonella enterica serotype Lubbock,* and *Trueperella pyogenes*	(72)
Grape pomace	Maceration (solvent: acid hydroalcoholic solution)	Caffeic Acid, Caftaric acid, Cutaric acid, Coumaric acid, Ferulic acid, Gallic acid, Syringic acid, Protocatechuic acid, Tyrosol, 4-Hydroxybenzoic acid, Vanillic acid, Naringenin, Isorhamnetin, Kaempferol, Myricetin, Quercetin, Laricitrin, Syringetin, Luteolin, Apigenin, Astilbin, Catechin, Epicatechin, Phloretin, Phlorizin, Resveratrol, Viniferin, Piceatannol, Astringin, Petunidin, Delphinidin, Peonidin, Cyanidin, Malvidin, Pelargonidin, Vitisin, Pinotin A	Bacterial growth inhibition assay	*E. coli*	(33)
Sun-dried grape pomace	Maceration Solvent: deionized water	n.d	Microdilution method	*L. monocytogenes, Staphylococcus aureus, Salmonella enterica subsp., enterica serovarm, Enteritidis,* and *Escherichia coli*	(7)
Grape pomace	Maceration Solvent: methanol	n.d	Antimicrobial activity assay under dynamic contact conditions	*Escherichia coli* and *Listeria innocua*	(51)
Grape: pruning firewood, stems, and lees	Maceration Solvent: methanol 70%	n.d	Disk diffusion method	*Acinetobacter baumannii, Pseudomonas aeruginosa, Staphylococcus aureus, Escherichia coli*	(73)
Grape pomace	Maceration (solvent: hexane)	Malvidin 3-glucoside, quercetin-3-O-glucoronide, kaempferol-3-O-glucuronide	Disk diffusion method	*Staphylococcus aureus, Escherichia coli, Aspergillus niger, Penicillium digitatum*	(70)
Grape (including skins, seeds and stems)	Maceration Solvent: 40:50:10 acetone:water:ethanol	Gallic acid, (+)-Catechin, (−)-Epicatechin, Quercetin-3-*O*-glucoside,	Disk diffusion method	*Staphylococcus aureus SVB-B13, Escherichia coli, Saccharomyces cerevisiae, Sclerotinia sclerotiorum*	(27)
Grape stalks	Maceration Solvent: water, acetone, diethyl ether, methanol and ethanol.	n.d	Microdilution plate method	*Bacillus subtilis, Bacillus cereus, Staphylococcus aureus, Pseudomonas aeruginosa, Escherichia coli,* and *Salmonella Enterica, Lactobacillus plantarum, Bifidobacterium animalis subsp. lactis and Bacillus subtilis, Staphylococcus aureus, Pseudomonas aeruginosa, Proteus mirabilis,* and *Escherichia coli, Rhodotorula mucilaginosa, Penicillium italicum, Penicillium expansum and Penicillium chrysogenum, Saccharomyces boulardii, Candida albicans, Trichoderma viride, Aspergillus flavus, Aspergillus fumigatus* and *Aspergillus niger*	(74)
Grape pomace	Maceration Solvent: water, ethanol 50%, ethanol 70% and acetone 50%, and acetone 70%	Caffeic Acid, Gallic Acid, Rosmanic Acid, Catechin, Epicatechin, Procyanidin, Kaempferol, Quercetin, Resveratrol	Modified hydrogel disk diffusion assay	*Staphylococcus aureus*	(65)
Grape pomace skins, pulp, stems and seeds	Decoction, microwave-assisted decoction, and soxhlet extractions Solvent: 60% MeOH/H2O	Kaempferol,. Resveratrol	Microdilution method	*Staphyloccocus aureus, Staphylococcus epidermidis, Escherichia coli,* and *fungus:* *Candida albicans*	(11)
Grape pomace	Reflux extraction Solvent: ethanol 50%	Gallic acid, Protocatechuic acid, Caftaric acid Gentisic acid, Catechin, Vanillic acid, Syringic acid, Epicatechin, p-Coumaric acid, Hyperoside, Isoquercitrin Rutin, Quercitrin, Quercetin, Luteolin	Disk diffusion method	*Streptococcus mutans, Porphyromonas gingivalis, Enterococcus faecalis, Escherichia coli, Staphylococcus aureus, Klebsiella* sp., and *Citrobacter* sp.,	(71)
skins and residual pulp, seeds and whole grape pomace	Ultrasound-assisted extraction/ maceration Solvent: etanol 50%	Gallic acid, tannic acid, chlorogenic acid, caffeic acid, p-coumaric acid, ferulic acid, rosmarinic acid, catechin, epicatechin, isoquercetin, naringin, myricetin, luteolin, quercetin, and naringenin.	Well diffusion assay/ Microdilution Technique	*B. cereus, Enterococcus faecalis, Ent. faecium, Ent. hirae, Listeria innocua, L. ivanovii, L. monocytogenes, Staphylococcus aureus, S. aureus, S. epidermidis, S. epidermidis, Streptococcus pyogens, Rhodococcus equi, Escherichia coli, Pseudomonas. aeruginosa, Salmonella enterica serovar Enteretidis, Salmonella enterica serovar Typhimurium,* and *Serratia marcescens, C. albicans, C. parapsilosis, C. glabrata, and C. tropicalis*	(4)
Syrah Grape Skin Residues	Ultrasound-Assisted Extraction (UAE) Solvent: ethanol 80%	caftaric acid; caffeic acid; gallic acid; Cis-resveratrol; Trans-resveratrol; Viniferin; (+)-Catechin; Procyanidin B1 and B2; Kaempferol-3-Oglucoside; Quercetin-β-Diglucoside; Isorhamnetin-3-glucoside-chloride; Myricetin; Rutin; Malvidin-3-glucoside-chloride; Cyanidin-3-glucoside-chloride; Pelargonidin-3-glucoside-chloride; Delfinidine-3-Oglucoside; Peonidine-3-Oglucoside	Microdilution Technique	*Staphylococcus aureus* and *Escherichia coli*	(5)
Grape seeds	Ultrasound-assisted extraction (Solvent: water)	n.d	Agar Well Diffusion Method	*Salmonella typhi, Bacillus cereus, Staphylococcus aureus, Escherichia coli, Candida albicans,* and *Aspergillus niger*	(67)
Grapes seeds from *Vitis vinifera* L	Ultrasonic extraction solvent: methanol-ethanol mixture (volume ratio of 1:1)	Gallic acid, Protocatechuic acid, *p*-Hydroxybenzoic acid, Chlorogenic acid, Caffeic acid, Syringic acid, Vanillin, *p*-Coumaric acid, Ferulic acid, Sinapic acid, Benzoic acid, *p*-Coumaric acid, Rosmarinic acid, Cinnamic acid, Catechin, Epicatechin, Hesperidin, Eriodictyol, Quercetin, Luteolin, Kaempferol, Apigenin	Agar well diffusion assay	*P. aeruginosa; Bacillus cereus; Enterococcus faecalis; Staphylococcus aureus; Methicillin-Resistant Staphylococcus aureus, Enterobacter aerogenes;* and *Escherichia coli*	(24)
Grape seed	Ultrasound-assisted extraction solvent: methanol	n.d	Agar well diffusion method	*Staphylococcus aureus, Escherichia coli*	(20)
Grape stalk	Ultrasound-assisted extraction Solvent; water and ethanol 70% or 80%/ Supercritical extraction	PF: Derivate of ferulic acid; 1,3-Dicaffeoylquinic acid; Gallic acid hexoside; Derivative of p-coumaric acid; Protocatechuic acid hexoside; Derivate of syringic acid; Proanthocyanidin dimer; Malvidin-3-O-glucoside; Proanthocyanidin dimer; Proanthocyanidin dimer; Malvidin-acetyl-hexoside; Proanthocyanidin trimer; Syringic acid, Vanillin; (+)-Catechin; Proanthocyanidin dimer, Rutin; Quercetin-3-O-galactoside; Quercetin-3-O-glucuronide; Syringetin-3-O-glucoside; Quercetin; Trihydroxyflavone; Trihydroxyflavanone; 3-Hydroxy-3′-methoxyflavone; 3′,4′-Dimethoxy-7-hydroxyflavone; 3′,7-Dimethoxy-3-hydroxyflavone; Dihydroxyflavanone/pinocembrin *Terpenoids:* Corosolic acid and Abscisic acid	Microdilution assay	*Staphylococcus aureus, Bacillus cereus, Listeria monocytogenes, Listeria innocua, Zygo sacharomyces bailii, Candida boidinii*	(1)
Grape pomace: grape seeds, leaves and stalks	Ultrasound-assisted extraction Solvent: Ethanol 50%/Pressurized solvent extraction method and	Caftaric acid, Quercetin-3-*O*glucuronide and Quercetin-3-*O*glucopyranoside, Q-3-*O*rutinoside, k*-3-O-glucoside,* k*-3-O-rutinoside*	Agar-based fungal growth inhibition assay	*Aspergillus flavus, Aspergillus carbonarius, Fusarium verticillioides, Fusarium graminearum, Alternaria alternata*	(28)
Grape pomace	Ultrasound-assisted extraction Solvent: water	Benzoic acid, 3-Hydroxybenzoic acid, 4-Hydroxybenzoic acid 3,4-Dimethoxyphenylacetic acid, p-Coumaric acid, Gallic acid Ferulic acid, Caffeic acid, Sinapic acid, delphinidin 3-*O*-glucoside, cyanidin 3-*O*-glucoside, malvidin 3-*O*-glucoside Procyanidin, catechin, epicatechin, quercetin 3-*O*-galactoside kaempferol 3-*O*-glucoside, quercetin, kaempferol	Disk diffusion method	*Escherichia coli* and *Bacillus subtilis*	(91)
Grape skin	Ultrasound-assisted extraction (Solvent:water)	n.d	Simulated migration assay	*–*	(75)
Grape pomace	Ultrasound-assisted extraction Solvent: water or ethanol 50%	n.d	Agar well diffusion method/microdilution method	*Staphylococcus aureus, Escherichia coli, Bacillus cereus* and *Salmonella enterica* subsp. *Enterica, B. cereus* and *S. typhimurium*	(53)
Grape stalks	Subcritical water extraction	n.d.	Microdilution Method	*Listeria innocua* and *Escherichia coli*	(92)
Grape by-product	Vacuum-expansion technologies	n.d.	Disk diffusion assay and microdilution method	*Klebsiella pneumonia, Escherichia coli, P. putida, K. pneumoniae, B. cereus,* and *S. aureus*	(49)
Grape pomace	Deep eutectic solvent (DES).	Delphinidin 3-*O*-glucoside, Cyanidin-3-*O*-glucoside, Malvidin 3-*O*-glucoside, Petunidin 3-*O*-glucoside, Gallic acid, Protocatechuic acid, *p*-Hydroxybenzoic acid, Coutaric acid, Caffeic acid*, Epicatechin, Syringic acid, *p*-Coumaric acid, (+)-Catechin, Quercetin-3-*O*-glucoside, Quercetin-3-*O*-rutinoside, Kaempferol-3-*O*-glucoside, Quercetin	Disk diffusion technique	*Escherichia coli* and *Bacillus subtilis*	(25)
Grape pomace	Supercritical extraction	Isorhamnetin-3-*O*-glucoside; Kaempherol-3-*O*-glucoside; Quercetin-3-*O*-glucoside; Quercetin-3-*O*-glucuronide; Epicatechin; yanidin B, Catechin	Colorimetric and Spectrophotometric Assays	*Staphylococcus aureus*	(52)
Grape pomace	Supercritical fluid extraction	Gallic acid, Chlorogenic acid, Ferulic acid, Kaempferol, Quercetin	Agar Well Diffusion Assays	*Escherichia coli, Pseudomonas fluorescens, Staphylococcus aureus, Bacillus subtilis, Aeromonas hydrophila, Listeria monocytogenes* and *Aspergillus niger*	(29)

#### 4.1.1 Traditional methods

Traditional extraction techniques such as maceration are frequently used. In this method, plant material is soaked in a solvent—typically water or hydroalcoholic mixtures—under specific temperature and agitation conditions (65, 68, 72). For example, El-Sawi et al. (36) used 80% ethanol to macerate stems, seeds, and peels of *Vitis vinifera*, reporting the highest TPC and TFC levels in stem extracts. Krasteva et al. (21) evaluated seed extracts from various grape cultivars using 70% aqueous ethanol under magnetic stirring, reporting the highest TPC in Pinot Noir. Costa et al. (73) assessed methanol/water extracts from pruning firewood, stems, and lees of six native *Vitis vinifera* varieties, with the Jaen variety showing high levels of ortho-diphenols and flavonoids, and notable antioxidant and antimicrobial activity. Teixeira et al. (65) examined the effect of water, ethanol/water, and acetone/water on extraction efficiency from red and white grape pomace, finding water yielded up to 41% in white grape pomace and acetone up to 70%. Radojević et al. (74) evaluated various solvents (water, acetone, diethyl ether, methanol, ethanol) for polyphenol extraction from grape stalks via maceration. Ethyl acetate yielded the highest total phenols (60.08 mg GAE/g extract), while acetone resulted in the highest total flavonoids (34.24 mg RUE/g extract). Acetone extracts also exhibited the strongest antimicrobial activity, particularly against Gram-positive bacteria, attributed to their phenol and flavonoid content. Díaz et al. studied the effects of maceration time and agitation on polyphenol extraction from Carménère, Cabernet Sauvignon, and Merlot grape pomace. They found that 12 h of extraction using 0.5 g of GP yielded the highest total phenolic content (TPC) and antioxidant activity, with no significant improvement beyond this duration. Among the three varieties, Merlot presented the highest TPC (70.36 ± 1.75 mg GAE g^−1^ GP extract), followed by Carménère (60.86 ± 7.49 mg GAE g^−1^) and Cabernet Sauvignon (57.03 ± 4.78 mg GAE g^−1^). In terms of phenolic compound profiles, Carménère extracts exhibited the highest total anthocyanin (35.13 ± 1.13 μg eq/g), flavonol (3.56 ± 0.14 μg eq/g), and flavanol (7.32 ± 0.13 μg eq/g) contents. Merlot followed with 24.10 ± 1.13 μg eq/g anthocyanins, 1.88 ± 0.11 μg eq/g flavonols, and 6.70 ± 0.07 μg eq/g flavanols. Cabernet Sauvignon had the lowest anthocyanin content (10.94 ± 0.36 μg eq/g), and flavonols (2.17 ± 0.28 μg eq/g), while flavanols were below the detection limit (23).

Other conventional methods such as decoction and Soxhlet involve boiling the plant material in the solvent (7, 11, 71) compared Soxhlet extraction, MAE, and decoction using 60% methanol/water. Soxhlet yielded the highest efficiency and polyphenol content. Pop et al. (71) prepared extracts from *Vitis vinifera* by-products by refluxing at 80°C with 50% ethanol, with HPLC analysis revealing distinct polyphenolic profiles.

Despite methodological differences, maceration remains the most widely used technique. Optimizing parameters such as duration and concentration enhances phenolic recovery. Soxhlet and reflux methods, while effective, are less common due to longer durations and higher solvent use.

#### 4.1.2 Advanced extraction technologies

UAE enhances solvent penetration and mass transfer via acoustic cavitation. This is the most frequently applied non-traditional technique (4, 5, 24, 28, 53, 67, 75). Andrade et al. applied UAE to Syrah grape skin residues, obtaining high TPC ranging from 196 to 733.7 mg·GAE/100 g, and a total flavonoid content between 9.8 and 40.0 mg·QE/100 g (5). Grosu et al. (4) combined UAE with maceration and demonstrated that grape seed extracts from the Romanian cultivars *Tămâioasă Românească* and *Fetească Neagră* yielded the highest phenolic and flavonoid contents (5). Specifically, TPC values reached 90.43 ± 0.50 mg GAE/g and 93.52 ± 3.06 mg GAE/g for the two cultivars, respectively. Total flavonoid content also peaked in seeds, with 86.86 ± 7.24 mg RE/g in *Fetească Neagră.* Giorni et al. evaluated UAE and pressurized liquid extraction (Naviglio®) and found that grape seed UAE extracts yielded the highest TPC (514.26 g GAE/100 g dry extract) among 14 extracts from 10 food by-products (28). In contrast, flavonoid content was highest in grapevine leaf extracts obtained using the NAV technique, with 126.41 g hyperoside/100 g dry extract, double the flavonoid content of grape seed UAE. Sateriale et al. varied UAE parameters, finding the highest TPC at 50°C, 40 kHz for 15 min or 20 kHz for 30 min using distilled water (53).

Mollica et al. studied MAE of grape skins, pulp, and stems, comparing results to decoction and Soxhlet. The latter two methods showed higher efficiency (43%) than MAE (26%). Soxhlet extraction of grape skin yielded the highest polyphenol content, antioxidant activity, and enzyme inhibition (11).

SFE using supercritical CO₂ has been explored for extracting bioactives from grape residues. Mihalcea et al. (29) tested different SFE conditions on Cabernet pomace, finding increased TPC with higher temperature and pressure. Verano-Naranjo et al. (52) noted that solvent polarity affects extract composition. Casquete et al. (1) compared UAE and SFE for grape stalks, reporting that UAE yielded phenolic-rich extracts with strong antioxidant activity, while SFE recovered more lipophilic compounds like terpenoids and fatty acids. De Freitas et al. (92) evaluated subcritical water extraction, which offers a green alternative for bioactive recovery.

DES are being investigated as sustainable solvents. Vorobyova et al. (25) used DES to extract polyphenol-rich grape pomace extracts, later incorporated into silver nanoparticles with notable antimicrobial activity.

Moreno-Chambas et al. (49) employed vacuum expansion to extract phenolics and anthocyanins from grape pomace. This technique involves rapid decompression following high-pressure heating, which disrupts cellular structure, increases porosity, and enhances solvent penetration—resulting in significantly improved extraction efficiency.

Despite the growing interest in grape pomace as a source of bioactive compounds, the literature on phenolic extraction reveals substantial methodological heterogeneity, posing significant challenges for direct comparisons of total phenolic and flavonoid content across studies. Most reported results, pertain to total extracts (e.g., TPC and TFC values). However, where available, data for individual phenolic components are identified, and detailed information can be found in Table 1. An extensive review of recent publications demonstrates that TPC values vary widely, even when similar raw materials are used (76, 77). This variability arises from multiple factors, including the type of matrix (e.g., skins, seeds, stems, or whole pomace), pre-treatment steps (e.g., drying, milling), and the extraction technique and conditions employed (78, 79).

Extraction methods differ greatly in solvent type and concentration (e.g., aqueous ethanol, methanol, acetone), solid-to-liquid ratio, temperature, duration, and equipment. For instance, while conventional maceration may yield moderate TPC values, pressurized liquid extraction has reported yields as high as 78.85 g GAE/kg dry weight, outperforming traditional solvent-based methods (77). Even among modern techniques like ultrasound-assisted extraction (UAE) and microwave-assisted extraction (MAE), the final phenolic yield is highly sensitive to operational parameters such as ultrasonic frequency, microwave power, and temperature (78). Moreover, the recent adoption of green solvents such as NADES has introduced further variability, with TPC values ranging from 5.94 to 43.73 mg GAE/g depending on the formulation and conditions (78, 79). Together, these methodological discrepancies—combined with the intrinsic variability of grape pomace—undermine the feasibility of reliable quantitative comparisons across the literature.

### 4.2 Antimicrobial activity assays

According to the literature reviewed, as detailed in Section 2, the most extensively studied bacterial genera are *Escherichia*, *Staphylococcus*, and *Bacillus*, followed, in decreasing order of frequency, by *Salmonella*, *Pseudomonas*, and *Listeria*. Within these genera, the most commonly analyzed species are *E. coli*, *S. aureus*, *B. subtilis*, *S. enterica*, *P. aeruginosa*, and *L. monocytogenes*, respectively (Table 1). The most commonly employed methodologies for evaluating the antimicrobial activity of GP extracts include the micro- or macrodilution methods, agar well and disk diffusion assays, and a variety of colorimetric and spectrophotometric techniques. These methods often provide complementary information and are used in combination in several studies (4, 24, 80). Based on the reviewed literature, these methodologies can be broadly classified into two categories: those that yield quantitative data and those that are qualitative in nature. Table 1 summarizes the methodologies employed by each author to assess the antimicrobial activity of grape pomace extracts or their derivatives, as well as the microbial strains used for these determinations.

#### 4.2.1 Macro- or microdilution methods

These methods are widely employed to determine the Minimum Inhibitory Concentration (MIC), defined as the lowest concentration of extract capable of inhibiting microbial growth (18, 53, 81). For example, Goulas et al. (7) reported that infusions derived from “Xinisteri” GP inhibited Listeria strains at concentrations equivalent to a typical cup of infusion (MIC = 0.25–0.5 mg/mL), demonstrating the synergistic effects of GP phytochemicals. This methodology is also employed to determine the Minimum Bactericidal Concentration (MBC)—the lowest concentration required to achieve a ≥ 99.9% reduction in the initial bacterial population—and the Sub-Minimum Inhibitory Concentration (SMIC) (81). Moreover, the Minimum Fungicidal Concentration (MFC) has been assessed using these approaches in some studies (5).

#### 4.2.2 Diffusion methods

These assays evaluate bacterial growth inhibition by measuring the diameter of inhibition zones. It is noteworthy that diffusion methods are frequently combined with microdilution techniques to determine the MIC (4, 80).

The agar well diffusion assay has been widely used (14, 20, 60, 66). For instance, Grosu et al. (4) assessed the antimicrobial potential of extracts from Fetească Neagră and Tămâioasă Românească grape varieties using the agar well diffusion method. The extracts were tested against 18 pathogenic bacteria and 4 *Candida* spp., showing high antimicrobial activity particularly against Gram-negative strains such as *Pseudomonas aeruginosa a*nd *Serratia marcescens*. They also exhibited pronounced inhibitory effects (diameters >10 mm) against *Staphylococcus*, *Enterococcus*, *Listeria*, *R. equi*, and moderate activity (6–10 mm) against *Bacillus cereus* and *Enterococcus* spp.

The disk diffusion technique is also frequently employed (27, 49, 69–71, 73). Modifications, such as the use of hydrogel-based disks, have been reported (65). Inga et al. (70) applied this method to assess the antimicrobial activity of GP extracts against *Staphylococcus aureus* (ATCC 25923), *Escherichia coli* (ATCC 25922), *Aspergillus niger* (061471010), and *Penicillium digitatum* (SSFC 36296), reporting inhibition zones of 7.95 ± 0.15 mm and 7.9 ± 0.1 mm for *Staphylococcus aureus* and *Escherichia coli*, respectively.

#### 4.2.3 Colorimetric and spectrophotometric assays

These methods are used for indirect quantification of microbial viability or biomass. Some studies have used Total Viable Count (TVC) and optical density (OD) at 600 nm (50, 52). Da Silva et al. (50) used UV–Vis spectrophotometry to assess bacterial growth inhibition in films incorporating GP extracts. Their results demonstrated antimicrobial activity against *E. coli* and *Bacillus subtilis* and suggested the potential application of such films in active food packaging due to their low water vapor permeability.

Giorni et al. (28) conducted fungal growth inhibition assays on agar media using grape seed, stalk, and other extracts. Gómez-Mejía et al. (2) employed a colorimetric assay based on p-iodonitrotetrazolium chloride (INT), which is reduced by metabolically active cells to red-colored formazan, allowing for visual or spectrophotometric detection. Verano-Naranjo et al. (52) used a microtiter plate assay based on the reduction of 2,3,5-triphenyltetrazolium chloride (TTC) to assess bacterial viability. TTC is reduced by cellular dehydrogenases to a red formazan, providing an indirect measure of metabolic activity.

#### 4.2.4 Advanced methods

As a part of gene expression analysis, the ability of aqueous and ethanolic grape seed extracts to inhibit the expression of the sea gene encoding staphylococcal enterotoxin A in *S. aureus* isolates was investigated (81). Bacterial cultures treated with the MIC of the extracts underwent RNA extraction, followed by cDNA synthesis and qPCR analysis of toxin-related gene expression. The expression of the sea gene was significantly downregulated (by 0.31–0.63 fold) in all pathogenic and environmental isolates.

Regarding how these extracts can be used in food to prevent microbial contamination and spoilage, Qiu et al. (75) conducted a simulated migration assay to indirectly evaluate antimicrobial activity in a food matrix. A bioactive film incorporating grape skin extract was tested under milk storage conditions to assess its efficacy in delaying microbial spoilage through the release of antimicrobial compounds. Sofi et al. (82) applied the Surface Contact Antibacterial Activity Assay, a method that simulates contamination scenarios by exposing material surfaces directly to bacterial cells, reflecting real-world applications in food packaging and biomedical devices.

### 4.3 Encapsulation strategies

Various encapsulation strategies have been explored to enhance the antimicrobial efficacy and stability of GP extracts, particularly through nanoparticle-based systems and protein–polysaccharide matrices.

Vorobyova et al. (25) synthesized silver nanoparticles (AgNPs) using deep eutectic solvent (DES)-derived GP extracts, achieving antimicrobial activity against *Bacillus subtilis* and *Escherichia coli*, as confirmed via the agar well diffusion method, with inhibition zones of 9 mm and 16 mm, respectively. Similarly, Anitha et al. (20) reported the synergistic antibacterial and antioxidant potential of bioconjugated silver nanoparticles synthesized from a grape seed extract mixture and chemically reduced silver. The polyphenol–AgNP complexes exhibited potent bactericidal activity, highlighting their relevance in addressing antibiotic-resistant bacterial strains.

In the study from Selim et al. (67) developed green nanoparticles by combining grape seed extract with metallic selenium and zinc oxide. These hybrid nanoparticles aim to capitalize on the bioactivity of plant-derived polyphenols and the antimicrobial properties of metal oxides, presenting a sustainable alternative for food preservation or biomedical use.

The use of chitosan (CH) coatings enriched with water-soluble polyphenol extracts from grape seed was investigated by Zhao et al. (59). Their findings demonstrated that such coatings effectively inhibited the growth of spoilage bacteria, particularly *Pseudomonas* spp., and mitigated the deterioration of physical attributes including color, texture, and water-holding capacity in meat fillets. Fillets treated with composite coatings (CH + grape seed polyphenols) showed improved quality during storage compared to those coated with chitosan alone, indicating a synergistic antibacterial effect.

Mihalcea et al. (29) investigated the encapsulation of GP extracts using a coacervation technique involving two different formulations: one based on bovine *β*-lactoglobulin and lactose, and another with Maillard reaction-induced glycosylation. The resulting powders demonstrated high retention of polyphenols and flavonoids, with enhanced protective capacity observed in the Maillard-induced formulation. This study not only provided a detailed phytochemical characterization of a local grape variety but also highlighted the relevance of glycosylation in improving the stabilization and delivery of bioactive compounds for diverse applications.

## 5 Applications of grape pomace in food preservation

### 5.1 Applications of grape pomace in food as additive

Over the years, food preservation has become a major concern due to spoilage, which has led to continuous efforts to extend shelf life, reduce wastage and ensure food safety (83). Strategies employed include the replacement of synthetic food preservatives and the extensive use of antibiotics with natural antimicrobials (13). Grape waste, which is rich in bioactive compounds such as polyphenols, flavonoids and tannins, has shown great potential for extending the shelf life of food (42). Their antimicrobial and antioxidant properties make these extracts promising candidates for application in food preservation strategies (32, 49).

Investigating the antibacterial and antifungal activity of grape waste is important to discover new, effective, naturally occurring food preservatives. In addition, its use as a food additive can help improve human health by preventing lipid oxidation and the formation of potentially harmful compounds such as acrylamide and improving sensory properties such as color and texture (7, 36). Studies have shown that the addition of grape seed extract to cheese increases its shelf life and improves its antioxidant potential. It also inhibits the growth of psychrotrophic bacteria, molds and yeasts (84). Similar results were observed in yogurt samples supplemented with grape seed extract, which showed a significant effect on the inhibition of pathogens. In that case, correlations were found between the added concentration of the extract and antibacterial activity against *Escherichia coli* (Pearson’s *r* = 0.914; *p* < 0.05) and *Staphylococcus aureus* (Pearson’s *r* = 0.952; *p* < 0.05) (60).

In addition, the addition of red grape pomace to sheep cheese showed the potential to inhibit the growth of Pseudomonas (MIC = 25 mg/mL), a microorganism often associated with spoilage of food of animal origin. The cheese also showed an inhibitory effect (MIC = 50 mg/mL) against several foodborne pathogens, including *Escherichia coli*, *Listeria monocytogenes*, *Salmonella Typhimurium* and *Stenotrophomonas maltophilia* (19). In this context, the use of grape residues as antimicrobial agents for the development of innovative foods or the fortification of existing products has been studied in detail. Table 2 shows the use of these residues as antimicrobial agents in various foods.

**Table 2:** Main applications of grape by-products in food systems and their reported antimicrobial activities.

Food application	Source	Antimicrobial properties	Reference
Active packaging	Grape by-product after the wine process making	Presented antimicrobial activity against *S. aureus*	(48)
Food packaging	Grape pomace	Highest bactericidal effect against this Gram-negative bacteria (*Escherichia coli*) and remarkable antimicrobial response against *B. subtilis*	(50)
Bioactive paper packaging	Grape seed oil	Bacterial inhibition for both Gram-negative and Gram-positive bacteria	(64)
Active packaging	Grape seed	Decreased the growth rate of the microorganism of turkey breast meat	(93)
Active food packaging	Grape stalks	Antibacterial effect against *Escherichia coli* and *Listeria innocua*	(92)
Food packaging	Commercial seed extract	Increased antimicrobial activity against *Escherichia coli* and *Staphylococcus aureus*	(54)
Edible coating	Commercial grape seed extract	Antimicrobial activity against *Escherichia coli* and *L. monocytogenes* by delaying bacterial growth	(61)
Active film	Commercial grape seed extract	Inhibitori effect on *Escherichia coli, Staphylococcus aureus and bacillus subtilis*	(57)
Active biocomposite films	Grape pomace	Antimicrobial activity against *Escherichia coli* and *Listeria innocua*	(51)
Composite film	Grape skin	Film inhibited fungal growth	(75)
Biodegradable film	Red grape pomace	Film presented great antimicrobial properties against *Staphylococcus aureus*	(66)
Color indicator films	Red grape skin	Strong antimicrobial ability against both *Escherichia coli* and *Staphylococcus aureus*	(63)
Chitosan coating	Grape pomace powder	Inhibit bacteria growth, especially spoilage bacteria of Pseudomonas	(59)
Yogurt fortified	Red grape seed extract	Enhanced the antimicrobial action against different Gram-negative, Gram-positive bacteria, and fungi	(94)
Yoghurt fortified	Grape seed	Inhibitory effect on pathogens’ growth	(60)
Ovine Vastedda-like stretched cheese	Red grape pomace	Grape pomace powder showed a large inhibition spectrum against spoilage and pathogenic bacteria.	(19)
Ingredient for Herbal Infusion	Grape Pomace	Antimicrobial effects against *Listeria monocytogenes* serotypes and other common food pathogenic bacteria	(7)
Fish products	Commercial grape seed	Significantly reduced the relative abundance of proteobacterial in the early stage and changed the distribution of the microbial community in the later stage of storage	(58)
Pork Burgers	White wine pomace	Counts of the microorganisms tested were not affected by the addition	(46)
Fish products	Grape Seed extract	GSE was more efficient against *Staphylococcus aureus* and *Bacillus subtilis*	(82)
Biopreservatives on goat meat	Grape seed extract	Pseudomonadales, Bacillales and Flavobacteriales were effectively inhibited	(56)

### 5.2 Applications of grape pomace in food packaging

In connection with innovative food preservation technologies, scientific interest in smart packaging has increased in recent years. The incorporation of bioactive compounds into packaging has been explored as an effective strategy to extend the shelf life of products and ensure their quality during production, storage and marketing (83, 85). Smart packaging increases food safety by enabling real-time monitoring of food quality throughout the supply chain. They control factors such as temperature fluctuations, microbial spoilage, packaging integrity and freshness, helping to reduce food waste and curb foodborne diseases (83). Among these technologies, freshness indicators stand out as a smart packaging system that monitors and communicates food quality through color changes that indicate microbial growth and chemical changes during storage and/or transportation (83). Active packaging technology, on the other hand, integrates substances with antioxidant and/or antimicrobial properties directly into the packaging material. The bioactive effect of active packaging contributes to the preservation of food by reducing accelerated deterioration processes and improving its nutritional, microbiological and physicochemical quality, thus extending its shelf life (85).

**Figure 5:**
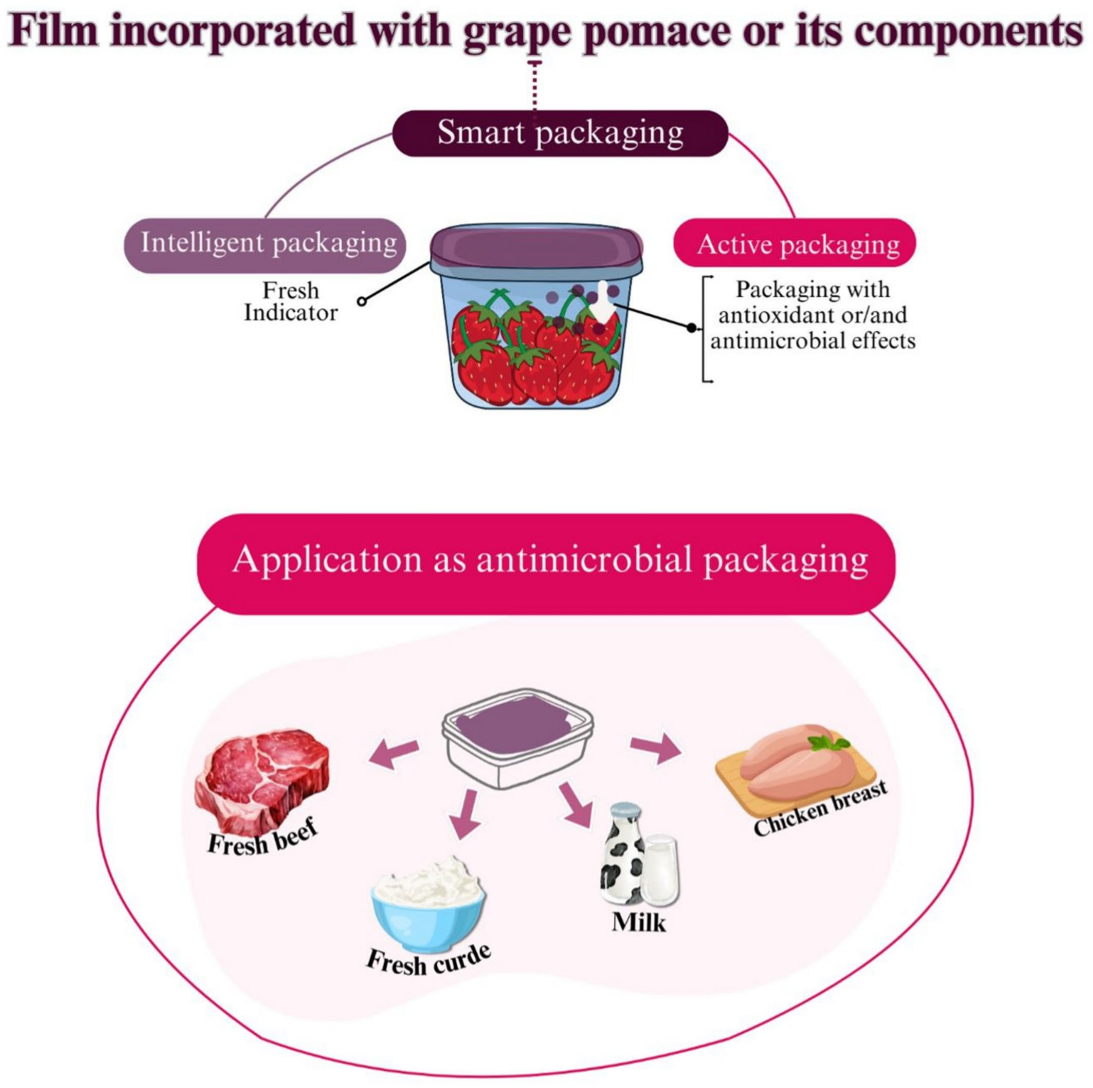
Smart packaging (active and intelligent) developed with the incorporation of grape pomace or its components and its application as antimicrobial packaging.

Originally, synthetic compounds were often used for this purpose. However, concerns about the potential health risks associated with these substances have encouraged the search for natural alternatives (85). In this context, grape waste is considered as a potential resource for natural pigments with halochromic properties, antioxidant and antimicrobial compounds in the development of smart food packaging (Figure 5). The overview focuses on the addition of grape pomace and its components to various films to improve their antimicrobial properties.

In a study by Moselhy et al. (80), coating with grape by-products had a significant effect on the microbiological preservation of chicken meat. During 21 days of storage at 4°C, the coated chickens showed a significant reduction in counts in the four microbial groups studied (total bacterial count, spore-forming bacteria, molds and yeasts and total coliforms). In contrast, in the chicken from the control treatment, the total bacterial count at the end of storage exceeded the safety index (80). Qiu et al. (75) investigated the antifungal activity of polyvinyl alcohol (PVA) films containing 15% grape skins (UGS15-PVA) and PVA films. Both films were immersed in milk to allow the polyphenols to transfer from the film into the liquid. It was observed that the milk that came into contact with the PVA film was completely covered with fungi. In contrast, the milk stored with the UGS15 PVA film showed significantly less fungal growth. This result suggests that grape polyphenols have an antifungal effect.

Compared to the control samples, the film-forming solutions with aqueous grape seed extract led to a 2.8-log_10_ and 5.5-log_10_ reduction in the viable count of *E. coli* (gram-negative) and *S. aureus* (gram-positive) (54). The antibacterial activity tests show that the addition of grape pomace extract gives the polypropylene films a bactericidal activity of over 30% against *Escherichia coli*. In the case of *Bacillus subtilis* (Gram-positive bacteria), this activity is over 50% for all films produced with different proportions of the extract. This bactericidal effect is due to the presence of polyphenols in the film with grape residues, which irreparably damage structural proteins and other important biomolecules such as DNA and RNA. Despite undergoing thermal treatment during processing, the films retain antimicrobial functionality due to the partial stability of polyphenols present in the incorporated grape pomace; a significant fraction of these compounds resists degradation and remains active within the film matrix (50).

Compounds extracted from grape pomace and incorporated into films also show antimicrobial activity. Bilayer films rich in grape tannins showed bacteriostatic activity against *Listeria innocua* and *Escherichia coli* (37). These results underline the potential of grape tannin films as a good alternative for active coatings or packaging material to control bacterial growth.

### 5.3 Patents reporting the application of grape pomace in food

To better understand the technological development and emerging trends in the industrial use of grape pomace for food purposes, patent data was retrieved from the Derwent World Patents Index (accessed March 13, 2025). The patents listed in Table 3 emphasize the use of grape pomace and its components, either as an antimicrobial agent, as a food additive or as an active component in food packaging. These innovations focus on the development of new functional food products, antimicrobial ingredients and active or edible packaging materials. In particular, the concentration of patents in countries such as China and South Korea indicates a strategic investment in the valorization of winery by-products through biotechnological and packaging innovations. In contrast, countries such as Brazil, despite being a major grape producer, still show limited technological patent activity in this area, highlighting a gap and a potential opportunity for innovation and industrial scaling in the Latin American context.

**Table 3:** Patents currently available in the electronic database Derwent World Patents Index to the food industrial applications of grape residue.

Patent N°	Title	Grape residue	Product/ingredient developed	Publication date	Country
CN119214270-A	Composite antioxidant functional food additive used in food used for preventing or delaying oxidation reaction of grease, comprises pomegranate peel extract, citrus peel extract, grape peel extract, and apple peel extract	Grape peel	Functional food additive has high antibacterial effect, which greatly protects the problem of food breeding bacteria, and effect of extending the shelf life of food.	December 31, 2024	China
CN118923792(A)	Composite bactericidal preservative used in food processing, is prepared by compounding grape seed extract, pomegranate seed extract and chitosan	Grape seed	Bactericidal preservative for food	November 12, 2024	China
CN118530619(A)	Aqueous ink comprises pigment, acrylic resin, solvent, dispersant, stabilizer, plant-based antibacterial agent, adhesion promoter and oil solvent useful for food package printing	Grape seed	Aqueous ink with antibacterial functionality for food package printing	August 23, 2024	China
CN117887219(A)	Degradable packaging film with secondary carrier carrying natural antibacterial agent is prepared by using preset amount of degradable matrix resin, secondary carrier, natural antibacterial agent, sustained release agent, and emulsifier	Grape seed	Biodegradable food packaging material with antibacterial functionality	April 16, 2024	China
KR2653695(B1)	Biodegradable food packaging material comprises paper, base layer formed by mixing copolymer resin, polyethyleneimine, calcium carbonate, tourmaline powder, modified graphene oxide and natural plant extract powder, adhesive layer, and coating layer	Grape seed	Biodegradable food packaging material with antibacterial functionality	April 03, 2024	Korea
KR2597930-B1	Mixed fermented powder used for, e.g., improving immunity and health, and suppressing obesity and anemia, comprises mixture of fermented mushroom powder, fermented grain powder, freeze-dried fruit powder, and dietary fiber powder	Grape peel	Mixed fermented powder consisting of fermented mushroom powder, fermented grain powder, freeze-dried fruit powder and dietary fiber powder (grape peel) has an antibacterial effect	November 02, 2023	Korea
KR2585695-B1	Preparing mixed aged powder useful for, e.g., treating high blood pressure and diabetes, reducing cholesterol, and enhancing immunity, by mixing aged mushroom powder, aged grain powder, freeze-dried fruit powder, and dietary fiber powder	Grape peel	Mixture of aged powder with aged mushroom powder, aged grain powder, freeze-dried fruit powder and dietary fiber powder (grape skin) has antibacterial effect	October 05, 2023	Korea
BR102022023852(A2)	Food mixture useful for preparing cakes, pies, cupcakes and muffins, contains sugar and/or light raisins, cassava flour, chemical yeast, cocoa powder, industrial residue of fermented or unfermented grape marc and fructooligosaccharide	Industrial residue of fermented or unfermented grape marc	Food mixture useful for preparing cakes, pies, cupcakes and muffins with antimicrobial effects	September 19, 2023	Brazil
ZA202210563-A	Preserving strawberries, used in field of food preservation comprises directly spraying chitosan-proanthocyanidin composite solution on strawberries seedlings, ventilating, drying and forming film	Grape seed	The composition is effective in extending the storage life of harvested strawberries and has antibacterial properties.	February 22, 2023	South Africa
CN114773866-A	Preparing edible film useful as food packaging material involves adding walnut protein peptide, glycerol in water to obtain film-forming liquid, adding modified plant extract, polyglutamic acid and composite antioxidant into film forming liquid, stirring and reacting, and degassing	Grape seed	Edible film, which is useful as a food packaging material, has a good antibacterial performance.	July 22, 2022	China
KR2022022743-A	Natural antibacterial composition, used in foods, cosmetics, pharmaceuticals, hygiene products, food packaging materials, comprises complex extract including a grapefruit extract, upper leaf extract and licorice extract, and golden extract	Grape peel	Natural antibacterial composition, used in foods and food packaging materials.	February 28, 2022	Korea
RO134733-A0	Preparing natural preservative used in food industry for sauces, by mixing grape pomace with hydroalcoholic solution, allowing to stand, decanting, filtering, cooling and centrifuging mixture, concentrating and/or drying extract	Grape pomace	Natural preservative with antimicrobial properties, which is used in food industry for vegetable oil rich sauces for preparing ready-to-eat salads.	February 26, 2021	Romania
CN111838296-A	Natural fresh-keeping preservative useful for sauce-stewed meat product, comprises grape seed extract, nutmeg extract, clove extract, tea polyphenol, pomegranate peel extract, and dehydrated vinegar powder	Grape seed	Natural fresh-keeping preservative has wide antibacterial range and significantly extends the shelf life of food.	October 30, 2020	China
JP2019149959-A	Producing shiitake mushroom extract useful for, e.g., preparing agent for improving storage life of food, by immersing shiitake in water solvent, and extracting	Grape skin/Grape seed	Shiitake extract and the component that improves shelf life (grape skin extract or grape seed extract) have an antibacterial and bacteriostatic effect.	September 12, 2019	Japan
BR102017022843-A2	Polymer film comprises byproduct of grape juice and sodium alginate, and is prepared by solubilizing glycerol per gram of alginate Sodium in distilled water with mechanical stirring, adding sodium alginate and product of grape juice	By-product of the grape juice	Polymer film has antimicrobial activity and increases shelf time of food.	May 07, 2019	Brazil
KR2018104518-A	Prebiotic used as, e.g., medicinal composition to promote proliferation of intestinal beneficial bacteria, e.g., *Lactobacillus kefiri* and inhibit growth of intestinal harmful bacteria, e.g., *Clostridium perfringens*, comprises grape seed flour	Grape seed	Prebiotic consisting of grape seed flour. The prebiotic is useful as a functional health food for promoting the proliferation of beneficial intestinal bacteria, and inhibiting the growth of harmful intestinal bacteria.	September 21, 2018	Korea
CN106617073-A	Health care product useful for inhibiting harmful bacteria, fungi and virus, comprises highly oxidized vegetable oil, normal temperature solid substrate and anhydrous additive	Grape seed	Health product is useful for inhibiting harmful bacteria, fungi, virus, and pathogen.	May 10, 2017	China
WO2016157029-A1	Preparation of stable phytocomplex comprising active antioxidant molecules starting from plant material by crushing plant material, water addition and mechanical micronization and dehydration of obtained aqueous suspension	Grape pomace	Powdered phytoderivative an ingredient or food additive with antimicrobial effect.	October 06, 2016	World Intellectual Property Organisation (WIPO)
US2016000094-A1	Composition used to inhibit growth of microbe in personal care formulation, and as preservative, e.g., in food products, comprises benzoic acid, botanical extract, essential oil, e.g., cinnamon oil, and alcohol solvent, e.g., phenyl ethanol	Grape seed	Composition used to inhibit growth of microbe and as preservative food products.	January 07, 2016	United States of America
US2015216918-A1	Dietary composition used for treating, e.g., breast cancer condition or symptoms in individual comprises mushroom including Agaricus blazei, fermented soy, and curcuminoid consisting of curcumin or demethoxycurcumin	Grape seed	Dietary composition in form of beverage, capsule, tablet, caplet, emulsion, suspension, syrup, solid dosage form, liquid dosage form, and/or food with antimicrobial effect	August 06, 2015	United States of America

A crucial prerequisite for the use GP in food preservation is, as with any other food additive, compliance with legal and toxicological requirements. The European Union has one of the most advanced legal frameworks for food by-products. According to Regulations (EC) No 178/2002 and No 2015/2283, the use of food by-products as natural additives requires prior authorization as a novel food. In addition, the EU has a strict waste management policy that includes fines for the improper disposal of solid residues. Nevertheless, there is increasing innovation in the valorization of by-products for higher value applications such as food packaging and functional ingredients (86).

To utilize phenolic-rich extracts in food, food supplements or packaging, EU regulations require compliance with food quality standards. These include validated hygiene protocols, microbiological testing and screening for allergens, mycotoxins, pesticides and heavy metals using advanced analytical techniques such as LC–MS/MS, real-time PCR and ELISA (87).

A comprehensive literature review shows no toxicology studies or established ADI values for GP as a whole. Most of the available toxicological data focus on grape seed extract (GSE), a specific fraction of GP obtained by targeted extraction procedures. Thus, studies on GSE have demonstrated safety and bioactivity in both *in vivo* and *in vitro* models (13, 69). However, these results cannot be directly extrapolated to the whole pomace matrix, which contains peels, pulp and stems and has a more complex chemical composition.

Furthermore, there are no official recommendations for the ideal phenolic intake levels associated with health benefits or defined safety thresholds for food applications. Nevertheless, Rubín-García reported that in adults, improvements in health markers, including reductions in body weight, blood pressure and fasting plasma glucose levels, were associated with a total polyphenol intake of approximately 259 mg/day per 1,000 kcal, consisting mainly of flavonoids (166 mg/day/1000 kcal) and phenolic acids (72 mg/day/1000 kcal) (88). Similar trends were observed in adolescents, but with a higher polyphenol intake (~683 mg/day), mainly flavonoids (~530 mg/day) and phenolic acids (~97 mg/day) (89).

## 6 Conclusions and future perspectives

This bibliometric review underlines the growing scientific interest in GP and its components as promising sources of natural antimicrobial agents for food applications. A steady increase in publications was observed from 2021 to 2023, peaking in 2023. This reflects the intensification of research efforts in response to the global demand for clean-label food preservatives and sustainable by-product utilization strategies. Despite a decline in publications in 2024, the momentum built up in recent years confirms the potential of GP as a natural food additive in line with the principles of food safety, bioeconomy and circular economy.

The wine industry, especially the top producers such as Italy, Spain and Portugal, produces huge quantities of GP, much of which remains underutilized. The data analyzed confirms that European research is strongly focused on the valorization of this by-product, which has promising implications for other grape-producing countries such as Brazil and Egypt. The use of GP at the local or regional level can be a strategic way to valorize agro-industrial by-products, support environmental sustainability and promote rural development.

The mechanistic studies examined indicate that the antimicrobial efficacy of GP is multifactorial. Its polyphenolic compounds exert selective inhibitory effects on foodborne pathogens through various mechanisms, including inhibition of quorum sensing, membrane disruption, enzyme deactivation and impairment of nutrient uptake. Remarkably, the antimicrobial activity of GP is often concentration-dependent and ranges from bacteriostatic to bactericidal, highlighting its versatility in different food systems. These findings not only reinforce the scientific basis for the inclusion of GP in food preservation strategies, but also highlight their potential for strain-specific and formulation-tailored antimicrobial applications.

The review also shows a transition in research from compositional analysis to functional and mechanistic assessments, signaling a maturation of the field. Extraction technologies have evolved from conventional maceration and solvent-based techniques to advanced, environmentally friendly methods such as ultrasound-assisted and supercritical fluid extraction, which improve the recovery of bioactives while aligning with sustainable processing goals. However, the standardization of extraction methods, the stability of bioactive compounds and the optimization of formulation strategies remain challenges - areas that should be further investigated.

The patent analysis underlines the commercial and technological importance of GP. Notably, China and Korea lead in innovation, while only Brazil and China appear in both scientific articles and patent records, suggesting that a stronger link between science and technology policies is needed in other grape-producing regions. Patent applications show applications of GP not only as antimicrobial agents, but also as functional additives and components in active packaging, indicating its multifunctionality and industrial potential.

Furthermore, the growing interest in smart packaging is in line with consumers’ desire for safer, preservative-free food and the pursuit of sustainability in a broader sense. GP compounds can offer dual functionality: antimicrobial activity and antioxidant protection, making them suitable candidates for incorporation into active and intelligent packaging materials for the development of smart packaging.

Future developments in this area should take into account the regulatory framework to ensure the safe use of grape by-products in food. Currently, there is limited harmonization of global regulations for the use of food processing by-products. Establishing clearer regulatory guidelines and promoting international harmonization will be crucial to enable the commercial adoption of applications from GP. In addition, linking industries that generate by-products, such as winemaking, with sectors that are able to utilize these raw materials is critical.

To fully exploit the potential of GP, future strategies should prioritize the optimization of extraction processes, the selection of grape varieties with high concentrations of bioactive compounds and comprehensive techno-economic assessments of production scalability. Moreover, conducting life cycle assessments is essential to ensure that industrial applications are developed and implemented in a truly sustainable way. Interdisciplinary approaches involving food science, analytical chemistry, materials science, microbiology and regulatory affairs will be essential for the transition from proof-of-concept studies to market-ready solutions.

## Data Availability

The original contributions presented in the study are included in the article/supplementary material, further inquiries can be directed to the corresponding authors.
